# Supplementary figures and images for: Development and validation of web-based dynamic nomograms predictive of disease-free and overall survival in patients who underwent pneumonectomy for primary lung cancer
Source: PeerJ. 2023 Aug 21;11:e15938. doi: 10.7717/peerj.15938 (PMC10448881; doi:10.7717/peerj.15938)

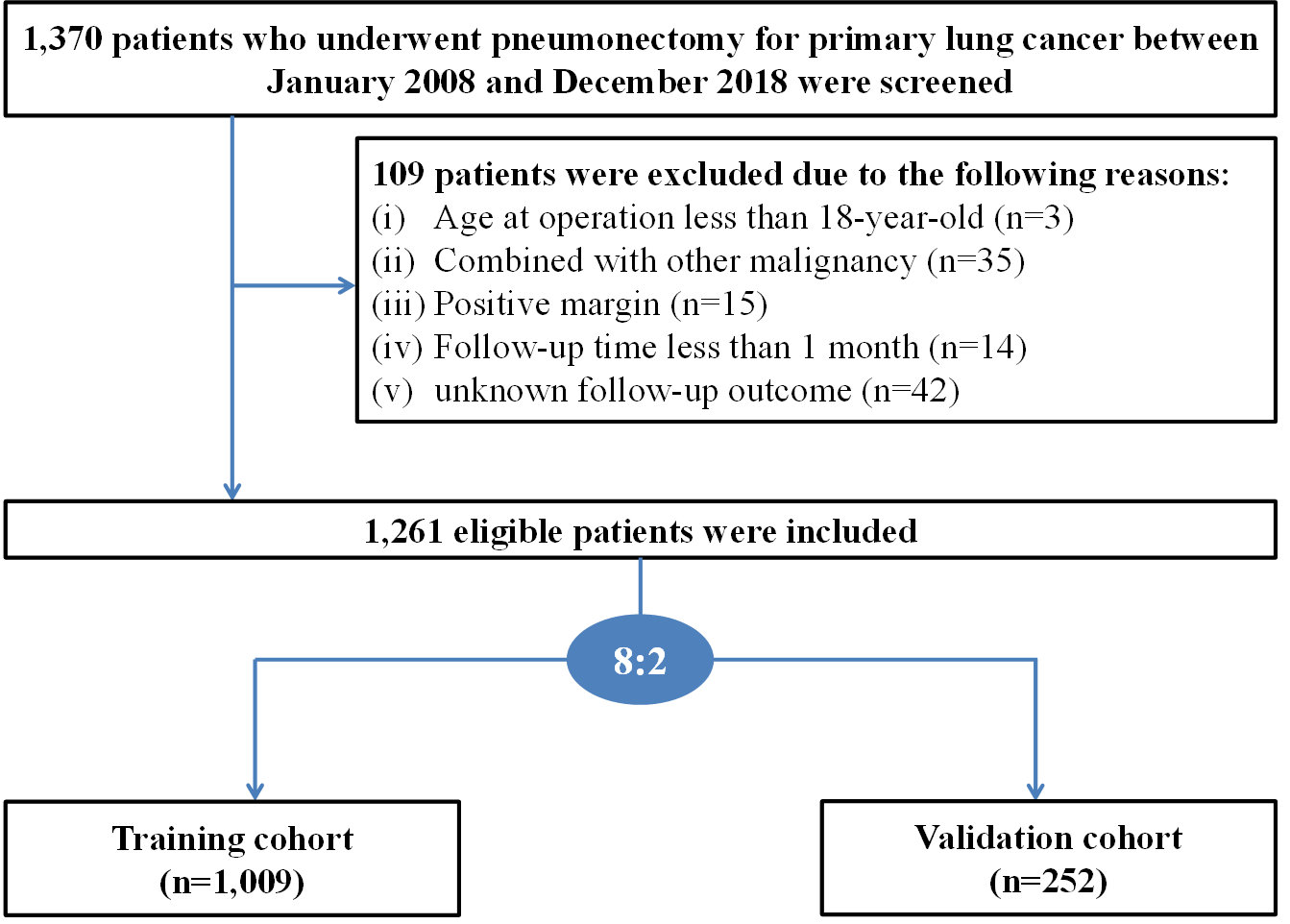

Supplement: Supplemental Information 1 [file peerj-11-15938-s001.tif]

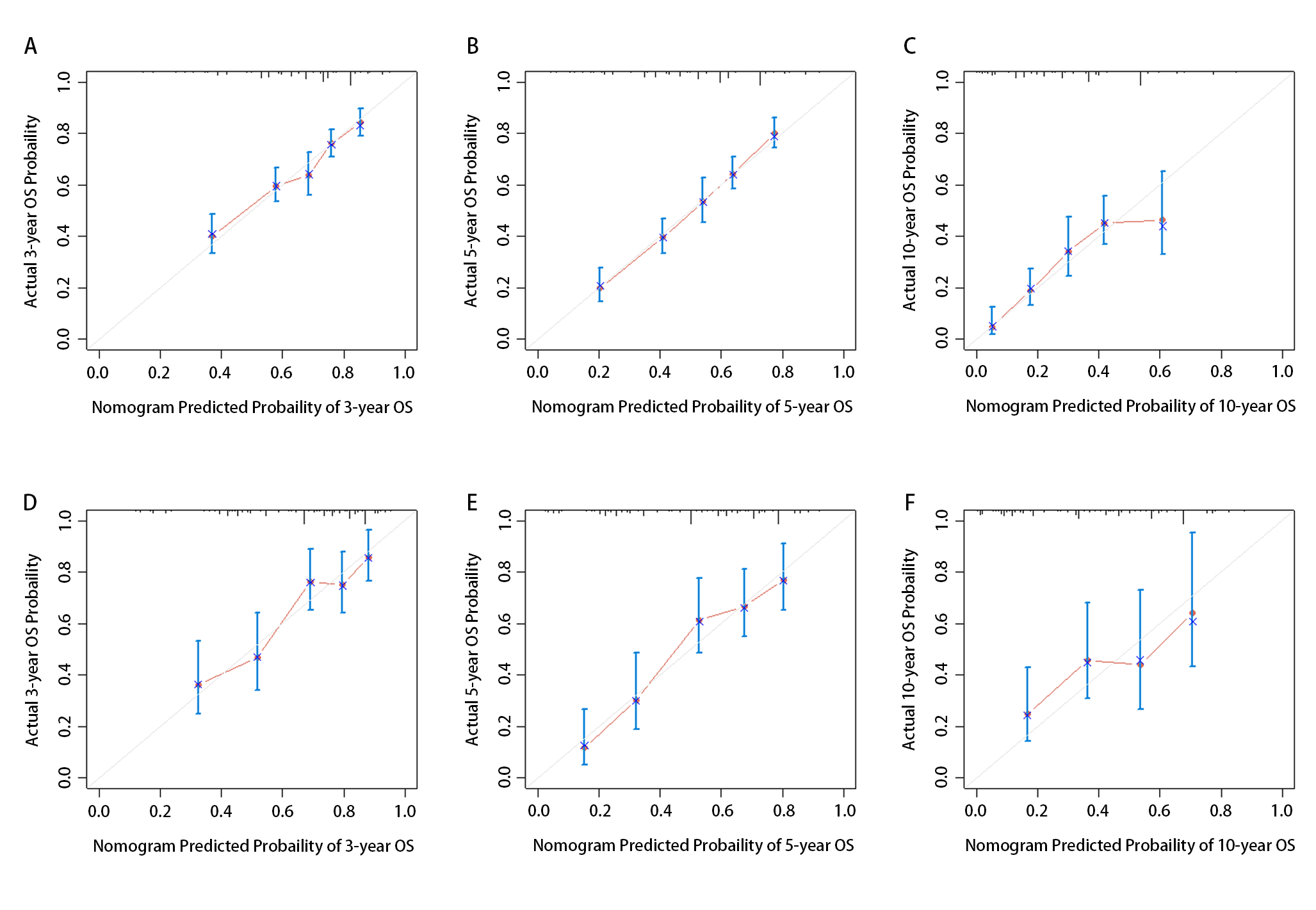

Supplement: Supplemental Information 2 [file peerj-11-15938-s002.png]

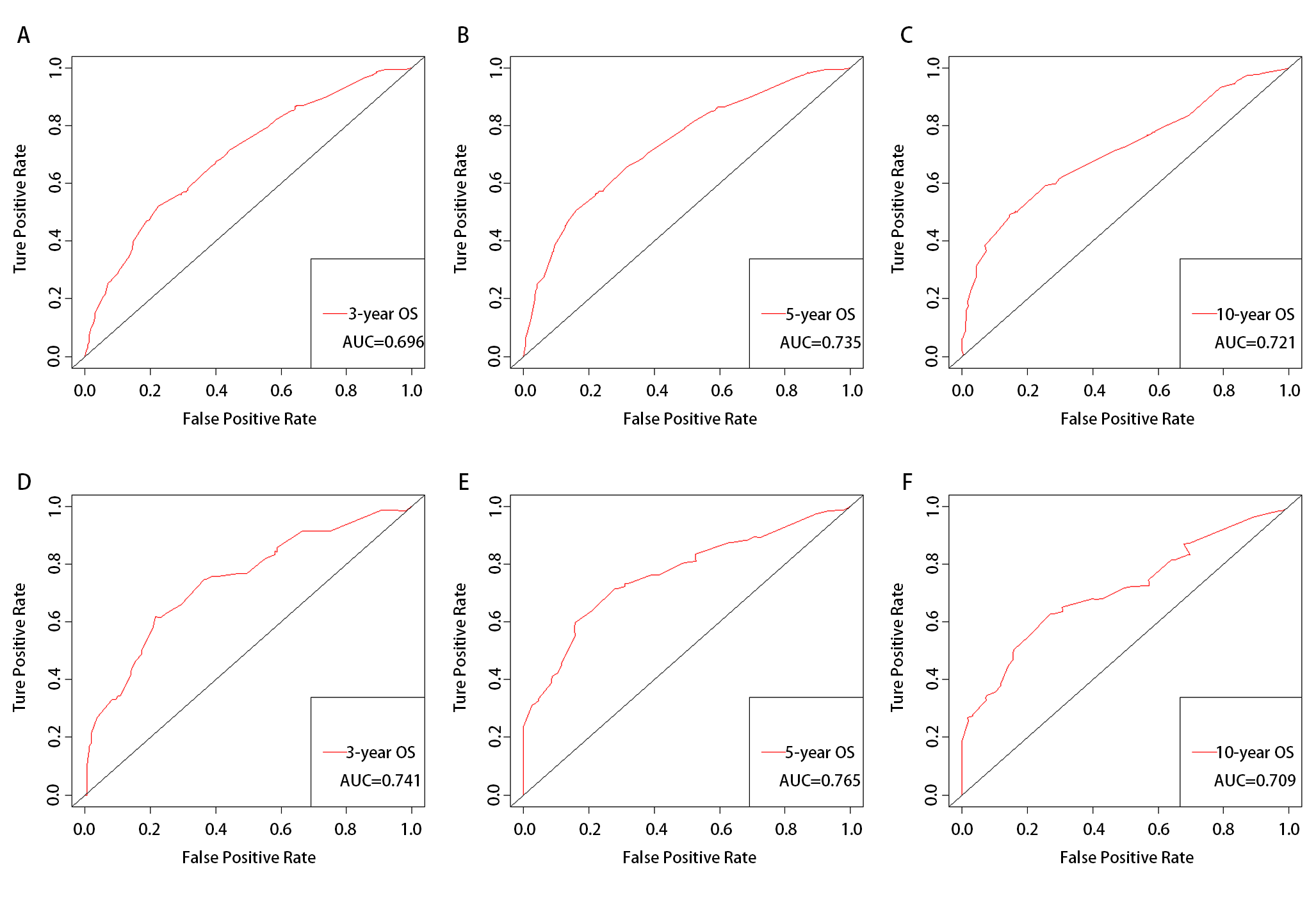

Supplement: Supplemental Information 3 [file peerj-11-15938-s003.png]

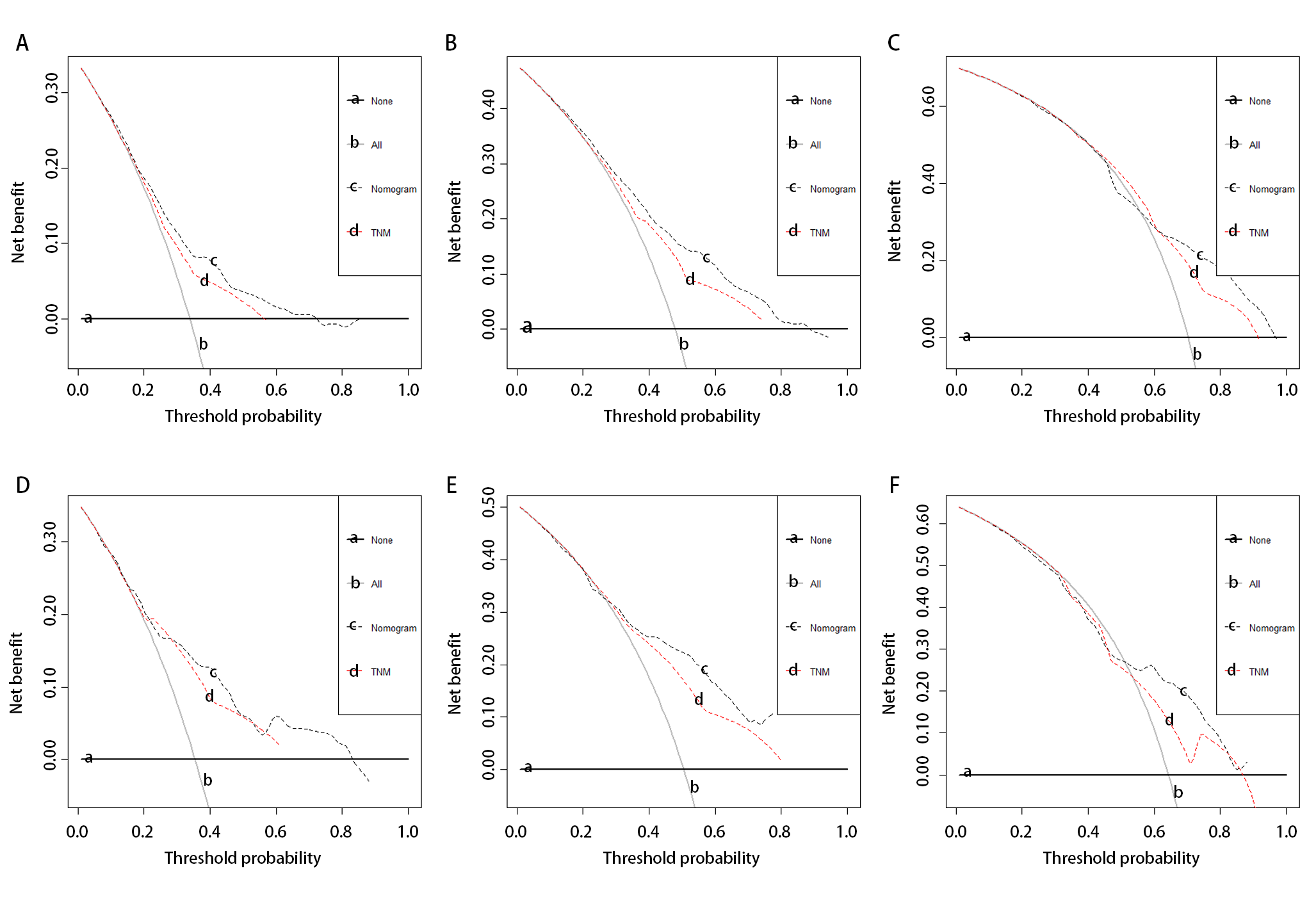

Supplement: Supplemental Information 4 [file peerj-11-15938-s004.png]
